# Vitamin D in Gambian children with discordant tuberculosis (TB) infection status despite matched TB exposure: a case control study

**DOI:** 10.1007/s00431-021-04272-z

**Published:** 2021-10-13

**Authors:** Lisa Stockdale, Basil Sambou, Muhamed Sissoko, Uzochukwu Egere, Abdou K. Sillah, Beate Kampmann, Robin Basu Roy

**Affiliations:** 1 Department of Paediatrics, University of Oxford, Oxford, UK; 2 NIHR Oxford Biomedical Research Centre, Oxford University Hospitals NHS Foundation Trust, Oxford, UK; 3 Vaccines and Immunity Theme, MRC Unit The Gambia, London School of Hygiene and Tropical Medicine, Atlantic Road, Fajara, The Gambia; 4 Department of International Public Health, Liverpool School of Tropical Medicine, Pembroke Place, L3 5QA Liverpool, UK; 5 Clinical Research Department, Faculty of Infectious & Tropical Diseases, London School of Hygiene and Tropical Medicine, Keppel Street, London, WC1E 7HT UK; 6 Division of Infectious Diseases and Tropical Medicine, Medical Centre of the University of Munich (LMU), 80802 Munich, Germany; 7 German Centre for Infection Research (DZIF), Partner Site Munich, 80802 Munich, Germany

**Keywords:** Tuberculosis, Vitamin D, The Gambia, TB, Paediatric, Case control

## Abstract

Using a matched case control design conducted at MRC Gambia in 2015, we measured vitamin D levels in pairs of asymptomatic children with discordant tuberculin skin test status despite the same sleeping proximity to the same adult TB index case. Median ages of groups (infected; 10.0 years, uninfected 8.8 years) were not significantly different (*p* = 0.13). Mean vitamin D levels were 2.05 ng/mL (95% CI − 0.288 to 4.38) higher in 24 highly TB-exposed uninfected children compared with 24 matched highly TB-exposed infected children (*p* = 0.08). The findings warrant further investigation in larger studies to understand the implications and significance.

* Conclusion*: Vitamin D levels were higher in TB-uninfected children compared with TB-infected despite equal high exposure to a TB case.

**What is Known:**

*• Paediatrics TB represents one of the leading causes of child death globally.*

*• Current literature shows an inconsistent relationship between vitamin D deficiency and increased risk of TB disease however a large Phase 3 trial of vitamin D supplementation in (largely vitamin D deficient) Mongolian children did not find any association with TB infection rates.*

**What is New:**

*• This study adds to the literature in a vitamin D sufficient paediatric population whereby children with equal exposure to a household TB case with no evidence of TB infection have higher levels of vitamin D compared with matched children with TB infection.*

## Introduction

Vitamin D deficiency has been associated with increased risk of tuberculosis (TB) disease 1, and infection 2; however, the definition of vitamin D deficiency is inconsistent. Host vitamin D receptor (VDR) genetic variants are also associated with risk of TB disease 3. Although vitamin D supplementation has recently been shown not to prevent latent TB infection in Mongolian children 4, vitamin D levels are influenced by genetics, cultural factors, diet, and latitude, making it unclear how these findings may relate to tropical African settings 5. Vitamin D is an immunomodulator with IFN-γ-dependent and IFN-γ and TNF-α-independent host mechanisms to control mycobacteria 6. Vitamin D-dependent antimicrobial peptides are hypothesised to down-regulate host pro-inflammatory responses responsible for lung pathology seen in TB disease 7. A negative tuberculin skin test (TST) or interferon-gamma release assay (IGRA) in an individual with a high risk of *Mycobacteria tuberculosis (M.tb)* exposure is likely evidence of elimination of the infection via innate immune responses or acquired immune response without T cell priming or memory 8. We hypothesised that vitamin D levels would be higher in highly TB-exposed uninfected Gambian children compared with matched highly TB-exposed infected children. We therefore measured vitamin D levels in stored samples from a 2015 study of pairs of children with discordant latent tuberculosis infection status despite matched exposure to the same household adult tuberculosis index case 9.

## Materials and methods

### Participant characteristics

As previously reported 9, pairs of asymptomatic children (5–15 years old) with discordant TST status despite equal sleeping proximity in the same building to the same adult index case with smear-positive pulmonary tuberculosis were recruited in the MRC Unit, The Gambia in 2015 (highly TB-exposed uninfected and ﻿highly TB-exposed infected children). Briefly, newly identified adult (> 15 years) smear positive TB cases were approached for consent. Children (< 15 years) within a cluster of the index case were consented for screening of TB symptoms and TST evaluation. Highly TB-exposed infected children were those with TST ≥ 10 mm. These children had an in-house IGRA test and were referred for further investigation for symptoms. All participants were screened for TB symptoms at three-monthly intervals for 1 year. Highly TB-exposed uninfected children were defined as TST negative (≤ 5 mm) at study initiation, and at a second TST 3 months after enrolment, despite exposure. All highly TB-exposed infected children were HIV negative, as were the adult TB index cases. Neonatal BCG vaccination in The Gambia is estimated at 98% 10; however, BCG vaccination status for participants was not collected.

### Inclusion and exclusion criteria

Smear positive index cases with more than two child contacts (between the ages of 5 and 15 years) were eligible for inclusion. After a valid TST had been placed, read, and recorded, only instances where at least one child had a TST ≥ 10 mm and at least one child had a TST ≤ 5 mm and parental or guardian consent had been given were included.

Children < 5 years were excluded from this study as they were prescribed isoniazid preventative treatment as recommended by the WHO. Children were excluded if they had previously received treatment for TB infection or disease, or if they showed TB symptoms, or had an abnormal chest radiograph.

### Sample procedures

Venous blood was collected and serum separated from lithium heparin vacutainer tubes (BD, Oxford, UK). Samples were stored at – 80 °C and shipped on dry ice prior to thawing for analysis.

### Vitamin D quantitation

Vitamin D (25(OH)D) in serum was measured using a commercial FDA-approved, validated enzyme immunoassay according to manufacturer instructions (Immunodiagnostic Systems, UK). Vitamin D levels in ng/mL were read from a standard curve. Vitamin D deficiency here is defined as < 10 ng/mL and insufficiency as < 20 ng/mL 11.

### Statistical analyses

Shapiro–Wilk test was used to assess data distribution. Paired students’ *t* test was conducted on the difference in ng/mL 25(OH)D within matched pairs. Wilcoxon’s matched pairs signed rank test was used for age comparisons. McNemars Χ^2^ test was used for sex comparisons. Analyses were conducted using GraphPad Prism.

## Ethical approval

The study was approved by The Gambia Government/MRC Joint Ethics Committee (SCC1405 and SCC1273) and the Imperial College Healthcare Tissue Bank (R13071).

## Results

Of the 58 children (29 pairs) recruited to the study, five pairs did not have sufficient stored serum volume for vitamin D measurement for both children in a pair. The remaining 24 pairs (48 children) were included in analyses (Table 1). Median ages did not significantly differ between infected (10.0 years) and uninfected (8.8 years; *p* = 0.13). There was no significant difference in the proportion of males to females in either group (children = 0.77). Four highly TB-exposed infected children had negative or indeterminate IGRA. No participants developed active TB disease during 12 months of follow-up.

**Table 1:** Summary of participant characteristics

Characteristic	Highly TB-exposed uninfected children	Highly TB-exposed infected children	*p*
*n*	24	24	-
Sleeping proximity to index case			
Same room	1	1	-
Same house	23	23	-
Median age years (IQR, range)	8 (6.5–12, 5–14)	10 (8–12, 6–14)	0.13^a^
Males number (%)	12 (50.0%)	13 (54.2%)	0.77^b^
HIV infection in child (*n* = 24)	-	0	-
Median initial TST result in mm (IQR)	-	18 (15–20)	0.00^a^
Median repeat TST result in mm (IQR)	0 (0)	-	-
Initial IGRA results (%)			
Positive	-	20 (83.3%)	-
Negative	-	3 (12.5%)	-
Indeterminate	-	1 (4.2%)	-
Mean vitamin D levels in ng/mL (range)	20.76 (12.6–29.7)	18.72 (10.9–28.6)	0.083^c^
Number (%) with vitamin D deficiency (< 10 ng/mL)	0	0	-
Number (%) with vitamin D insufficiency (< 20 ng/mL)	9 (37.5%)	15 (62.5%)	0.08^b^

**Fig. 1:**
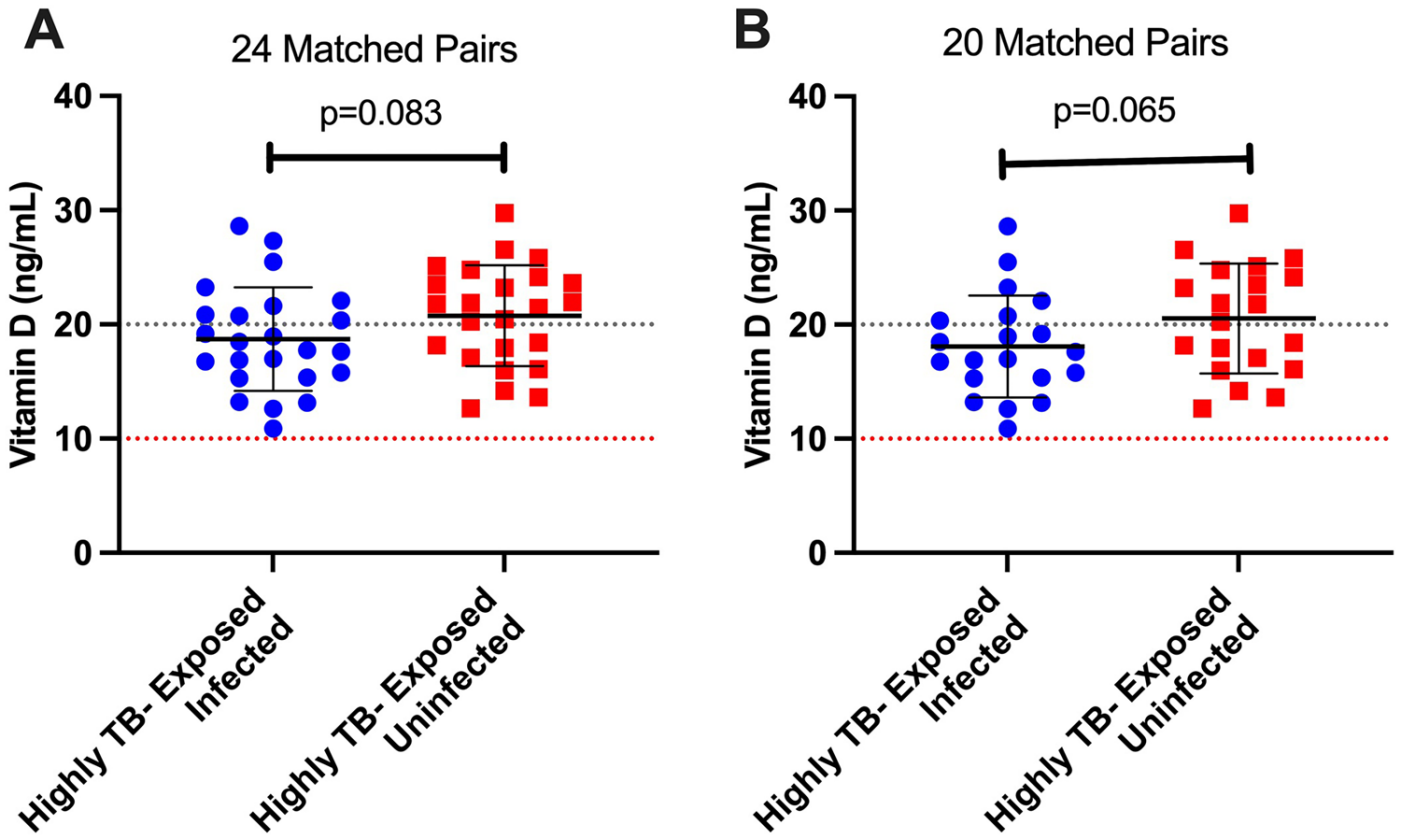
**A**&**B** Vitamin D levels among 24 matched pairs (**A**) and 20 matched (pairs subgroup analysis restricted to 20 pairs of children where the highly TB-exposed infected children had consistent IGRA/TST results at baseline, **B**) of highly TB-exposed uninfected and infected children. Red dotted line represents vitamin D deficiency threshold of 10 ng/mL; grey dotted line represents vitamin D insufficiency threshold of 20 ng/mL

^a^Wilcoxon’s matched pairs signed rank test, ^b^McNemars Χ^2^ test, ^c^paired Student’s *t* test.

Mean levels of vitamin D for 24 highly TB-exposed infected children was 18.72 ng/mL; range 10.9–28.6 ng/mL (46.8 nmol/L; range 27–71.5 nmol/L) and 24 highly TB-exposed uninfected children was 20.76 ng/mL; range 12.6–29.7 ng/mL (51.9 nmol/L; range 31.5–74.3 nmol/L), difference 2.05 ng/mL, 95% CI -0.288 to 4.38, p = 0.083; Fig. 1A). While no children included in this study were vitamin D deficient using the threshold of 10 ng/mL, 37.5% of highly TB-exposed uninfected children and 62.5% of highly TB-exposed infected children would be classed as insufficient, being below 20 ng/mL. The difference in proportions of vitamin D insufficient children is not significantly different between the two groups (*p* = 0.08, Table 1.)

As was conducted previously 9, a subgroup analysis was performed excluding the four pairs where the highly TB-exposed infected children had discordant IGRA/TST results. This subgroup analysis included 40 children (median ages 9.8 years and 9.1 years for infected and uninfected respectively; *p* = 0.44). Highly TB-exposed infected children had a mean vitamin D level of 18.08 ng/mL; range 10.9–28.6 ng/mL) (45.2 nmol/L; range 27.3–71.5 nmol/L), whereas the matched highly TB-exposed uninfected children had a mean level of 20.5 ng/mL; range 12.6–29.7 ng/mL (51.3 nmol/L; range 31.5- 74.3 nmol/L), difference 2.46 ng/mL, 95% CI -0.17 to 5.09, p = 0.065, Fig. 1B). All children (infected and uninfected) included in this study were above the threshold of 10 ng/mL (25 nmol/mL) for vitamin D deficiency (Fig. 1B).

## Discussion

Understanding paediatric protective immunity to acquisition of TB infection is vital in order to guide targeting of preventative and adjunctive therapy, vaccine design, and evaluation. Here, we compared vitamin D levels in latently infected children in a TB endemic country in children with a persistently negative TST despite matched household *M.tb* exposure. We identified a trend towards lower vitamin D levels in children with TB infection than those without evidence of infection; however, this did not reach significance. The trend was consistent in the subgroup analysis restricted to pairs of children where the highly TB-exposed infected children had consistent IGRA/TST results at baseline.

A 2020 household contact study in UK children found ﻿a stepwise decline in vitamin D levels from non-infected children to those with TB infection and then children with TB disease, with a significant difference between those with TB infection and TB disease 12. In a large study of 9810 Mongolian school children, Ganmaa et al. report an adjusted risk ratio of 1.23 ﻿[95% CI, 1.08–1.40], *p* = 0.002 for vitamin D deficiency, defined as < 10 ng/mL, and TB infection as determined by ﻿the QuantiFERON-TB (QFT) Gold assay 2. A subsequent phase 3 randomised controlled study in over 8,800 Mongolian children found that vitamin D oral supplementation over 3 years was not associated with any difference in QFT positivity, despite a mean increase of over 20 ng/mL vitamin D in the supplemented group 4.

A meta-analysis of ﻿3,544 (mainly adult) participants from 13 countries included in prospective trials investigating vitamin D and TB risk found a median vitamin D level of 26 ng/mL (﻿65.0 nmol/L; IQR 19.5–33.4 ng/mL) and a dose-dependent relationship between deficiency of vitamin D (< 10 ng/mL) and increased risk of incident TB, a finding which was significantly exacerbated by HIV 1. Another meta-analysis restricted to children < 18 years found that vitamin D deficiency was associated with TB with a pooled OR of 1.78 (95% CI 1.30–2.44, *p* < 0.05); however, the definition of vitamin D deficiency varied between < 30 ng/mL, < 20 ng/mL, and < 10 ng/mL in the studies included 13. The applicability of these findings to the Gambian population being studied here is not clear. While no Gambian children studied here would be classed as deficient using the 10 ng/mL (25 nmol/L) threshold for vitamin D deficiency, between 37.5% (highly TB-exposed uninfected) and 62.5% (highly TB-exposed infected) would be classed as insufficient by the thresholds used here. The Mongolian phase 3 study found that 31.8% of children had vitamin D levels below 10 ng/mL 4, and between 24% (of uninfected children) and 63% (of children with TB disease) included in the UK study were deemed vitamin D deficient using the same threshold 12.

While the trend between elevated vitamin D and absence of TB infection despite high TB-exposure reported here is consistent with results from existing literature 1, 12, a study in Gambian adults reporting higher serum vitamin D levels in adults with TB disease compared to household contacts 5 points towards variation in associations even within the same country.

Strengths of this study include the careful exposure-matched study design, comparing experimental data on samples from school-age children with TB infection to those who remain persistently uninfected despite defined household contact with an adult with smear-positive pulmonary tuberculosis. The original study was not powered to detect differences in vitamin D levels. Therefore, the small sample size of pairs of children from whom sufficient sample was available for this exploratory analysis is a limitation. IGRA status was available at baseline for the highly TB-exposed infected children but was not available for the highly TB-exposed uninfected children. Potentially confounding factors that may affect vitamin D levels (such as diet) are likely to be equally distributed within pairs living in the same household compound; therefore, the magnitude of vitamin D differences may be small.

Our data from this largely vitamin D-sufficient group of children with household tuberculosis exposure in a tropical African climate contribute to the body of evidence that higher vitamin D levels may be linked to lower risk of acquisition of TB infection. Larger studies utilising similar epidemiological designs in high TB-prevalence countries with distinct climates are required to further elucidate the connection between vitamin D levels and immunity against tuberculosis infection.

## Data Availability

All data will be made available.

## Abbreviations

IGRA: Interferon-gamma release assay
*M.tb*: *Mycobacteria tuberculosis*
TB: Tuberculosis
TST: Tuberculin skin test
VDR: Vitamin D receptor
